# Transmission reduction, health benefits, and upper-bound costs of interventions to improve retention on antiretroviral therapy: a combined analysis of three mathematical models

**DOI:** 10.1016/S2214-109X(22)00310-2

**Published:** 2022-08-09

**Authors:** Anna Bershteyn, Lise Jamieson, Hae-Young Kim, Ingrida Platais, Masabho P Milali, Edinah Mudimu, Debra ten Brink, Rowan Martin-Hughes, Sherrie L Kelly, Andrew N Phillips, Loveleen Bansi-Matharu, Valentina Cambiano, Paul Revill, Gesine Meyer-Rath, Brooke E Nichols

**Affiliations:** a Department of Population Health, New York University Grossman School of Medicine, New York, NY, USA; b Health Economics and Epidemiology Research Office (HE2RO), University of the Witwatersrand, Johannesburg, South Africa; c Department of Decision Sciences, University of South Africa, Pretoria, South Africa; d Burnet Institute, Melbourne, VIC, Australia; e Institute for Global Health, University College London, London, UK; f Centre for Health Economics, University of York, York, UK; g Department of Global Health, School of Public Health, Boston University, Boston, MA, USA; h Department of Medical Microbiology, Amsterdam University Medical Center, Amsterdam, Netherlands

## Abstract

**Background:**

In this so-called treat-all era, antiretroviral therapy (ART) interruptions contribute to an increasing proportion of HIV infections and deaths. Many strategies to improve retention on ART cost more than standard of care. In this study, we aimed to estimate the upper-bound costs at which such interventions should be adopted.

**Methods:**

In this combined analysis, we compared the infections averted, disability-adjusted life-years (DALYs) averted, and upper-bound costs of interventions that improve ART retention in three HIV models with diverse structures, assumptions, and baseline settings: EMOD in South Africa, Optima in Malawi, and Synthesis in sub-Saharan African low-income and middle-income countries (LMICs). We modelled estimates over a 40-year time horizon, from a baseline of Jan 1, 2022, when interventions would be implemented, to Jan 1, 2062. We varied increment of ART retention (25%, 50%, 75%, and 100% retention), the extent to which interventions could be targeted towards individuals at risk of interrupting ART, and cost-effectiveness thresholds in each setting.

**Findings:**

Despite simulating different settings and epidemic trends, all three models produced consistent estimates of health benefit (ie, DALYs averted) and transmission reduction per increment in retention. The range of estimates was 1·35–3·55 DALYs and 0·12–0·20 infections averted over the 40-year time horizon per additional person-year retained on ART. Upper-bound costs varied by setting and intervention effectiveness. Improving retention by 25% among all people receiving ART, regardless of risk of ART interruption, gave an upper-bound cost per person-year of US$2–6 in Optima (Malawi), $43–68 in Synthesis (LMICs in sub-Saharan Africa), and $28–180 in EMOD (South Africa). A maximally targeted and effective retention intervention had an upper-bound cost per person-year of US$93–223 in Optima (Malawi), $871–1389 in Synthesis (LMICs in sub-Saharan Africa), and $1013–6518 in EMOD (South Africa).

**Interpretation:**

Upper-bound costs that could improve ART retention vary across sub-Saharan African settings and are likely to be similar to or higher than was estimated before the start of the treat-all era. Upper-bound costs could be increased by targeting interventions to those most at risk of interrupting ART.

**Funding:**

Bill & Melinda Gates Foundation.

## Introduction

Sub-Saharan Africa is home to two-thirds of all people living with HIV. These individuals require lifelong treatment with antiretroviral therapy (ART) to safeguard their health and reduce HIV transmission.^1^ Expanded HIV testing and access to ART, including implementation of the so-called treat-all guidelines, same-day diagnosis, and ART initiation, has decreased the proportion of HIV-associated deaths^2^ and transmissions^3^ among ART-naive individuals. As a result, ART-experienced individuals, especially those who have interrupted ART, are contributing to an increasing proportion of HIV mortality and transmission in sub-Saharan Africa.^4^, ^5^ In response, HIV researchers and those implementing programme are investigating strategies to improve retention of people living with HIV on ART (appendix pp 1–11).

Among strategies that have been effective in improving retention, there are some have that have been implemented without incurring additional cost. An example is multimonth dispensing of ART, which showed improved retention in randomised trials in sub-Saharan Africa.^6^, ^7^ Adoption of multimonth dispensing was accelerated during the COVID-19 pandemic, and, in July, 2021, multimonth dispensing became part of WHO's Consolidated Guidance on HIV Treatment.

However, many other strategies that have been shown to be effective at improving retention incur additional cost.^8^ Examples include financial incentives,^9^ local delivery of medications,^10^ individual and group adherence support,^11^ viral-load-informed adherence counselling,^12^ and the use of mobile and wireless technologies to support retention (eg, mHealth services).^13^ Among these strategies, some are implemented for entire patient populations regardless of individual risk of treatment interruption, while others can be implemented targeting patients most at risk of treatment interruption, which usually reduces costs.

**Research in context**

**Evidence before this study**

Countries hard-hit by the HIV/AIDS pandemic in sub-Saharan Africa have made tremendous progress in expanding access to antiretroviral therapy (ART) since the implementation of the so-called treat all guidelines. As a result, an increasing proportion of AIDS-related deaths are now believed to occur among ART-experienced individuals who have interrupted treatment. To reduce AIDS-related deaths and transmission associated with ART interruption, retention interventions have been proposed, including multi-month dispensation, local delivery of medications, health worker and peer support, and mHealth services. Although some interventions, such as multi-month dispensing, are cost-neutral or cost-saving and accordingly are recommended by normative agencies, others incur added costs and it is not known when they should be implemented. We searched PubMed on Oct 17, 2021, for articles in English, with no date restrictions, using the terms (“upper-bound cost” OR “cost-effectiveness” OR “willingness to pay” OR “willingness-to-pay”) AND “retention” AND “Africa”. We identified one study done before the implementation of treat-all, which simulated a clinical cohort eligible to receive ART with CD4 counts of 350 cells per μL or lower, and estimated an upper-bound cost of US$10 per patient-year of improved retention for patients receiving ART. Because HIV incidence and mortality have decreased in the era of treat-all, there is concern that the amount countries would be willing to pay to improve retention might be even lower than previously estimated; however, these concerns could potentially be offset by the increasing contribution of ART interruptions to HIV mortality and transmission.

**Added value of this study**

This analysis presents the first estimation of the upper-bound costs of ART retention interventions in sub-Saharan Africa in the era of treat all. Using a multi-model comparison approach, our study takes into consideration a diversity of model structures, assumptions, and baseline settings to examine the robustness of upper-bound cost estimates to the sub-Saharan African setting and modelling methods. We found that the models agree regarding the cumulative health and transmission benefits of improving retention over a 40-year time horizon, but predict different kinetics of how quickly health and transmission benefits will accrue, resulting in more variable estimates at higher annual discounting rates. However, in most cases the upper-bound costs were greater than those estimated before treat all.

**Implications of all the available evidence**

Although HIV mortality and incidence have decreased in the treat-all era, upper-bound costs for improving retention remain similar or higher than previous estimates because of the important contribution of ART interruptions to continued HIV mortality and transmission. These findings should be encouraging to researchers investigating strategies to improve ART retention at added cost. Additionally, these results can aid decision makers in selecting available interventions for implementation, which might include targeting those most at risk of ART interruption.

Before the treat-all era, analyses suggested that an intervention for all people receiving ART that improves retention by 40% in could be cost-effective if it cost up to US$10 per person-year in low-income and middle-income countries (LMICs) in sub-Saharan Africa.^14^ However, this upper bound might have changed now that countries in sub-Saharan Africa have implemented the treat-all guidelines and greatly increased ART coverage. We aimed to estimate the transmission reduction, health benefits, and upper-bound costs to improve ART retention using three HIV transmission models based in sub-Saharan Africa with diverse structures, assumptions, and baseline settings, and using a range of economic benchmarks to assess upper-bound costs that could be spent to retain an additional person-year on ART.

## Methods

### Study design and model selection

In this combined analysis, we invited affiliates of the HIV Modelling Consortium to participate in our study if their models could provide annual projected estimates of incidence, prevalence, mortality, disability-adjusted life-years (DALYs), and ART coverage in a sub-Saharan African setting; include simulation of ART interruptions and their effect on transmission and mortality; and simulate a reduction in the rate of ART interruption by different degrees starting in 2022 and continuing until 2062. Three HIV epidemic models in the Consortium met these criteria and had diverse structures, assumptions, and baseline settings: EMOD, Optima, and Synthesis (table 1). Each model took a different approach to simulating the risk of transmission, morbidity, and mortality for ART-experienced people living with HIV who have interrupted ART use.

**Table 1:** Characteristics of the EMOD, Optima, and Synthesis HIV models

	**EMOD**	**Optima**	**Synthesis**
Setting	South Africa	Malawi	LMICs in sub-Saharan Africa
Model type	Individual-based	Compartmental	Individual-based
Transmission structure	Age-structured and sex-structured network for coital acts and childbirths	Force-of-infection for sexual (sex-structured) and vertical transmission	Viral load distribution in potential non-primary partners (according to age gender mixing) and primary partner
Untreated HIV disease progression in ART-naive individuals	Age-dependent rate of decline in CD4 count	Fixed rate of progression for each CD4 count category	Viral load changes over time (gradual increase), dependent on gender; CD4 count decline depends on latest viral load; AIDS rate depends on latest CD4 count, viral load, and age
Untreated HIV disease progression in ART-experienced individuals	Age-dependent progression rate starting at CD4 count when ART was interrupted	Same rate as for ART-naive individuals starting from CD4 count at ART interruption	Viral load increases to pre-ART level immediately, CD4 count moves towards pre-ART level gradually
Effect of ART	Recovery of CD4 cell count, suppression of viral load leads to reduced mortality and transmission	Recovery of CD4 cell count, suppression of viral load leads to reduced mortality and transmission	Recovery of CD4 cell count, suppression of viral load leads to reduced mortality and transmission
Baseline rate of ART interruption	Decreasing from 17·8% per year before so-called treat-all era to 3·4% per year by 2020	12·5% per year in 1990 decreasing to 4% per year by 2020 for all people on treatment; increasing from 23% to 29% per year from 2015 to 2019 among people with HIV with CD4 counts of <200 cells per μL, representing inconsistent treatment for those with previous ART interruption	Varies across setting scenarios and by factors including pregnancy, ART adherence, ART toxicity, and time on ART—eg, for a non-pregnant, ART-adherent individual with no ART toxicities, interruption rates range from 0·8% to 4·8% in the first year of ART
Baseline rate of ART re-initiation after interruption	Same rate as ART-naive individuals in the same population group (age, sex, CD4 count, AIDS symptoms, and pregnancy)	All ART-experienced individuals have an opportunity to re-link to care when they reach a CD4 count of <200 cells per μL	Varies across setting scenarios and by factors including pregnancy, sexual risk behaviour, and HIV symptoms

EMOD=EMOD-HIV. LMICs=low-income and middle-income countries. Optima=Optima HIV. Synthesis=HIV Synthesis.

EMOD-HIV, referred to hereon as EMOD, is an individual-based network transmission model of HIV calibrated to epidemic trends in South Africa.^15^, ^16^ In this model, the rate of progression of untreated HIV disease is assumed to be heterogeneous and age dependent. For example, for an individual infected at age 20 years, median survival time without treatment is estimated to be 13·1 years (IQR 8·4–18·5), whereas, for an individual infected at age 50 years, median survival time without treatment is estimated to be 6·3 years (4·1–8·9). In this model, during untreated chronic HIV infection, CD4 count decreases continuously on a square root scale, with median CD4 count of 507 cells per μL (IQR 398–613) 3 months after infection and 19 cells per μL (9–42) at time of AIDS-related death. While a person has viral load suppression on ART, transmission is reduced and CD4 cell count reconstitutes on a square root scale over the first 3 years, and then stabilises. ART interruptions result in resumption of untreated HIV progression on the basis of age and CD4 count at the time of interruption and return to pretreatment transmission potential. The rate of ART interruptions lasting more than 1 month was 18·7% per year before implementation of the treat-all guidelines and decreased to 3·4% per year by 2020.^17^ More details of EMOD are in the appendix (p 12).

Optima HIV, referred to hereon as Optima, is a compartmental HIV transmission model calibrated to epidemic trends in Malawi.^18^, ^19^ The model is disaggregated by sex, 5-year age group, and risk (female sex workers, clients of female sex workers, men who have sex with men, and general population). HIV progression is defined by category from acute infection (CD4 counts of ≥500, 350–499, 200–349, 50–199, and <50 cells per μL). CD4 cell count and viral load change at rates depending on ART use and latest reported CD4 cell count and viral load. In this model, ART use reduces transmission potential by 50% for unsuppressive ART and 100% for suppressive ART. Mortality both on and off ART depends on latest reported CD4 cell count and ART status (unsuppressive or suppressive), varying between 0·08% per year with a CD4 count of more than 500 cells per μL on suppressive ART to 32·3% per year for a CD4 count of less than 50 cells per μL not on ART. In the absence of retention programmes, ART interruption assumes that individuals would not return to care until a CD4 count of less than 200 cells per μL is reached via disease progression. The rate of ART interruption was assumed to be 12·5% per year in 2004, decreasing to 4% per year by 2020 for all people on treatment. For people living with HIV with a CD4 count of less than 200 cells per μL, the rate of ART interruption increased from 23% to 29% per year from 2015 to 2019, representing inconsistent treatment for those with previous interruption.^19^ Additional details of Optima are in the appendix (pp 12–13).

HIV Synthesis, referred to hereon as Synthesis, is an individual-based HIV model that tracks a simulated population of adults living in LMICs in sub-Saharan Africa.^20^, ^21^ HIV transmission is simulated between primary partners, and for non-primary partners, HIV acquisition risk depends on the viral load distribution among people of the opposite sex and in age categories determined by age–sex mixing patterns. The variables of ART regimen, ART adherence, and specific drug-resistance mutations jointly determine the antiviral effect of a regimen at any point in time. In this model, the benefit of ART is via its effect on viral load and CD4 count. ART interruptions cause an increase in viral load to pre-ART concentrations and a decrease in CD4 count towards pre-ART concentrations. ART interruption rates vary by setting scenario and by factors including pregnancy, ART adherence, ART toxicity, and time on ART. For example, for a non-pregnant, ART-adherent individual with no adverse events due to ART, interruption rates in the first year of ART range from 0·8% to 4·8% across setting scenarios.^22^ Additional details of Synthesis are in the appendix (pp 13–14).

### Model scenarios

Models simulated interventions that improve retention on ART beginning on Jan 1, 2022, and with outputs provided up to Jan 1, 2062, for a 40-year time horizon of intervention effects. Each model simulated interventions that, for all people on ART within the simulation, reduce the rate of treatment interruption by 25%, 50%, 75%, or 100% relative to the model's no-intervention baseline projection. We did a bounding analysis for the degree to which retention interventions could be targeted to people living with HIV most at risk of ART interruptions. Interventions were considered to be maximally targeted to those at risk of ART interruption if only incremental person-years on ART, added by the intervention, were counted towards intervention cost. Interventions were considered not to be targeted (ie, to be given to all people on ART regardless of risk of interruption) if all person-years on ART were counted towards intervention cost, including individuals who would have remained on ART in the absence of the intervention. For each scenario, standardised annual outputs were provided from each model including incidence and prevalence of HIV, number of people receiving ART, HIV-related deaths, and DALYs. All models calculated DALYs as the sum of years of life lost to HIV in each year of simulation, plus the years lived with treated and untreated HIV multiplied by respective disability weights from the 2017 Global Burden of Disease Study.^23^

### Analysis of model outputs

We compared epidemic trends (HIV incidence, prevalence, and mortality) from each model, and for each level of improvement in retention we assessed the health benefits and transmission reduction per additional person-year retained on ART. We did a bounding analysis for the extent that retention interventions can be targeted to individuals most at risk of having interrupted treatment. Using outputs for DALYs and new infections, for each model, we estimated the numbers of infections and DALYs averted for each additional person-year on ART relative to baseline, which we used to represent an intervention provided only to individuals who would otherwise have interrupted treatment, and for each person-year on ART regardless of baseline ART use, which we used to represent an intervention provided to all people on ART regardless of their risk of ART interruption. As a lower bound for targeting, we applied the incremental cost of the retention intervention to all people receiving ART, regardless of their risk of ART interruption. As an upper bound for targeting, we only applied the incremental cost of the retention intervention to the additional number of person-years on ART in the intervention scenario, relative to the baseline scenario. We report discounted costs and outcomes (ie, infections and DALYs) at the same rate (0%, 3%, or 6% per year). We inverted the ratios of DALYs averted to person-years on ART and number of infections averted to person-years on ART to calculate the number needed to treat (NNT).

We calculated the highest retention intervention cost at which net monetary benefit was positive—ie, at which incremental costs of the intervention were smaller than the product of DALYs averted multiplied by the cost-effectiveness threshold. Given uncertainty in cost-effectiveness thresholds, we calculated results for a range of thresholds and provide equations for calculating results with alternative thresholds in the appendix (pp 15–17). In Malawi and other LMICs in sub-Saharan Africa, we used a cost-effectiveness threshold range of US$500^24^ to $750^25^ based on cost-effectiveness at the margin of donor-financed HIV services, which generally exceeds the amount that could be afforded through domestic health-care expenditure alone. For South Africa, where HIV services are primarily domestically funded, we used a range of cost-effectiveness thresholds from $590 per DALY averted (based on opportunity cost at the margin of the South African HIV programme^26^) to $3525·12 per DALY averted (based on opportunity cost at the margin of all South African domestic health-care expenditure^27^). We accounted for the effects of retention on ART coverage by incorporating an annual ART cost of $206·75 in South Africa^28^ and $165·50 in Malawi^29^ and LMICs in sub-Saharan Africa,^30^ in addition to the cost of the retention intervention (appendix p 15). We discounted costs and DALYs at 0%, 3%, or 6% per year. All costs are reported in 2019 US$.

We did all analyses using R (version 4.0.3) and Microsoft Excel 2016.

### Role of the funding source

The funder had no role in the study design, data collection, data interpretation, data analysis, or writing of the report.

## Results

**Figure 1:**
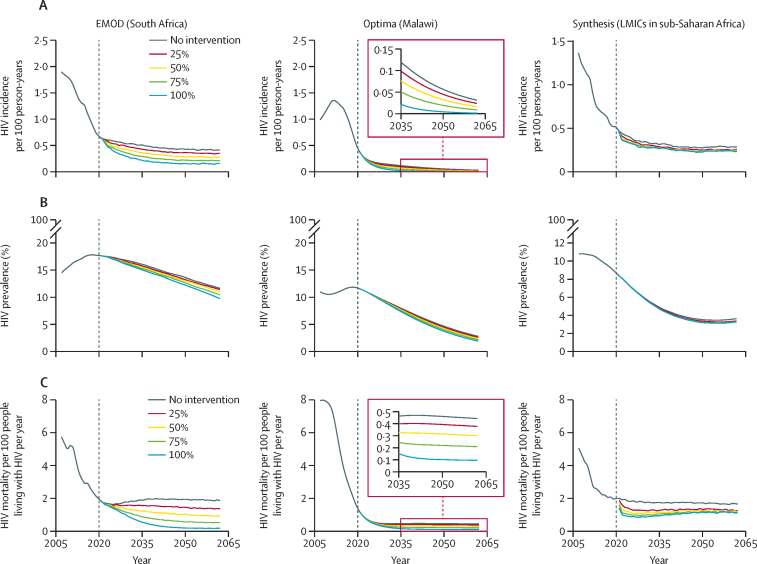
Projections of HIV incidence (A), prevalence (B), and mortality (C) with improvements to ART retention EMOD, Optima, and Synthesis model projections of HIV incidence per 100 person-years among adults aged 15 years and older (A), HIV prevalence among adults aged 15 years and older (B), and HIV deaths per 100 people living with HIV per year (C). Graphs show baseline projections with no intervention to improve ART retention and improved retention so that treatment interruption rates decrease by 25%, 50%, 75%, or 100% at the start of 2022 (grey vertical dashed lines). ART=antiretroviral therapy. EMOD=EMOD-HIV. LMICs=low-income and middle-income countries. Optima=Optima HIV. Synthesis=HIV Synthesis.

The EMOD, Optima, and Synthesis models produced different epidemic patterns (figure 1; appendix p 17) reflecting their diverse model structures, assumptions, and the different epidemic patterns in the settings being modelled. Baseline HIV incidence was highest in Synthesis and lowest in Optima. Baseline HIV prevalence was similar in EMOD and Synthesis and lower in Optima. Baseline HIV mortality rates were similar in the EMOD and Synthesis models and lower in Optima (figure 1). For all three models, increasing ART retention reduced HIV prevalence, incidence, and mortality, with the largest decreases seen in EMOD (figure 1). Kinetics of the response of the HIV epidemic to improved retention varied widely across models, reflecting the variety of ways in which HIV transmission and disease progression dynamics were modelled. Synthesis manifested the most front-loaded response, with incidence and mortality decreasing immediately upon improvement in retention in 2022. 100% retention in 2022 was predicted to reduce mortality among people living with HIV in Synthesis by 51·7% in 2023 compared with the baseline scenario with no change in retention. By contrast, under the same scenario, Optima projected the most delayed response, with the same intervention reducing mortality by only 3·9% in 2023, but with substantial reductions in mortality over the 40-year time horizon of analysis. EMOD projected a decrease in mortality of 6·4% in 2023 compared with baseline under the same scenario, from 1·6 AIDS-related deaths per 100 people living with HIV in 2022 to 0·2 deaths per 100 people living with HIV in 2062, but showed the largest decreases in mortality over the 40-year time horizon of analysis (appendix p 17).

**Figure 2:**
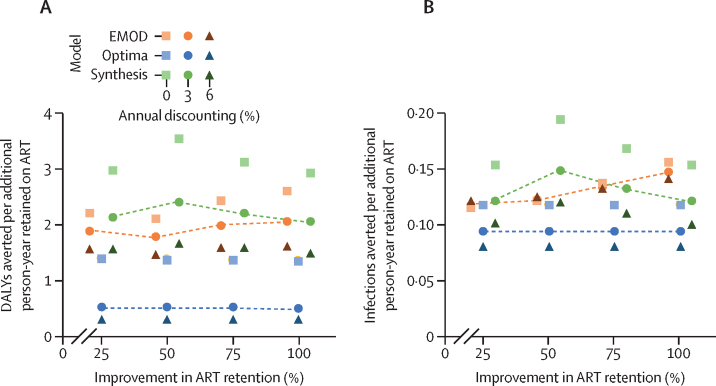
Health benefits (A) and transmission reduction (B) per additional person-year retained on ART with improved retention, representing a maximally targeted retention intervention Model estimates from EMOD, Optima, and Synthesis showing the ratios of DALYs averted (A) and HIV infections averted (B) per additional person-year retained on ART, with annual discounting of 0%, 3%, and 6%, at different levels of improvement in ART retention (25–100%). Inverting these numbers provides estimates of the number needed to treat, where the number treated is the additional number of individuals on ART compared with the no intervention scenario (ie, those who would have interrupted ART without improvement to retention). ART=antiretroviral therapy. DALYs=disability-adjusted life-years. EMOD=EMOD-HIV. Optima=Optima HIV. Synthesis=HIV Synthesis.

In all models, health benefits, in the form of DALYs averted, arose both from the direct reduction in mortality among people living with HIV who were better retained on ART, and from the avoidance of further HIV infections through maintenance of viral load suppression (figure 1; appendix pp 1–11). Despite wide variation in epidemic patterns, all models produced similar estimates of health benefit and transmission reduction per additional person-year on ART. Health benefits, measured in DALYs averted per person-year on ART, were consistent among models, robust to the degree of improvement in retention, and consistent among models with no discounting, but more variable with 3% and 6% annual discounting (figure 2A). Without discounting, all models and retention levels produced estimates within a factor of two of each other, ranging from 1·35 (Optima) to 3·55 (Synthesis) DALYs averted per person-year retained on ART. Discounted at 3% per year, these estimates spanned a factor of four, from 0·52 (Optima) to 2·41 (Synthesis) DALYs averted per person-year retained.

Estimates of number of HIV infections averted per additional person-year retained on ART were similar across models, levels of improvement in ART retention, and discount rates (figure 2B). Undiscounted estimates ranged from 0·12 (EMOD) to 0·20 (Synthesis) infections averted per additional person-year on ART, resulting in an NNT range of 4·9–8·2 additional person-years needed to be retained on ART to avert one HIV infection. Discounted at 3% per year, estimates ranged from 0·10 (Optima) to 0·16 (Synthesis) infections averted per additional person-year on ART, resulting in an NNT range of 6·4–10·1 additional person-years needed to be retained on ART to avert one HIV infection.

**Figure 3:**
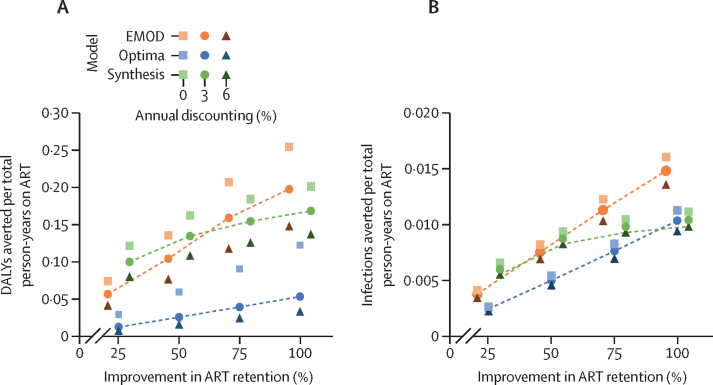
Health benefits (A) and transmission reduction (B) per total person-years on ART with improved retention, representing a minimally targeted retention intervention Model estimates from EMOD, Optima, and Synthesis showing the ratios of DALYs averted (A) and HIV infections averted (B) to total person-years on ART with annual discounting of 0%, 3%, and 6%, at different levels of improvement in ART retention (25–100%). Inverting these numbers provides estimates of the number needed to treat, where the number treated is the total number of individuals on ART, regardless of whether or not the intervention changed the retention status. ART=antiretroviral therapy. DALYs=disability-adjusted life-years. EMOD=EMOD-HIV. Optima=Optima HIV. Synthesis=HIV Synthesis.

The number of infections averted and health benefits per total person-years on ART varied widely across models (figure 3). As expected, increased improvements in retention resulted in an increase in effect per person-year on ART in all models because all individuals on ART were considered to have received an intervention regardless of whether retention status changed as a result. Synthesis projected the largest effect per person-years on ART: at 50% improvement in retention with 3% discounting, Synthesis projected 0·135 DALYs and 0·009 infections averted per person-year on ART, yielding an NNT of 7·4 to avert one DALY and 114·1 to avert one infection. Optima predicted the smallest effect per person-year on ART: at 50% improvement in retention with 3% discounting, Optima projected 0·026 DALYs and 0·005 infections averted per person-year on ART, yielding an NNT of 37·8 to avert one DALY and 199·4 to avert one infection. Estimates from EMOD were between those of the other models: at a 50% improvement in retention with 3% annual discounting, EMOD projected 0·105 DALYs and 0·008 infections averted per person-year on ART, yielding an NNT of 9·5 to avert one DALY and 120·9 to avert one infection. Results were less sensitive to discount rate and more sensitive to the model used and the level of improvement in ART retention.

Ranges of upper-bound costs that could be spent to cost-effectively retain an additional individual on ART (table 2; appendix pp 15–17) implied substantial variability by setting and model projection. Upper-bound cost was highest with EMOD because of a combination of larger health benefits from improving retention and a higher cost-effectiveness threshold used for South Africa than in the other model scenarios (table 2). Upper-bound costs that could be spent per additional person-year retained on ART was lowest for projections with Optima because of smaller health benefit from improving retention and a lower cost-effectiveness threshold used for Malawi than for other model scenarios (table 2).

**Table 2:** Upper-bound costs to improve ART retention

	**Retention interventions for people most at risk of ART interruption**			**Retention interventions for all people on ART**		
	EMOD	Optima	Synthesis	EMOD	Optima	Synthesis
25% improvement in ART retention	90–5919	99–232	910–1448	28–180	2–6	43–68
50% improvement in ART retention	851–5624	97–228	1039–1641	50–329	5–12	58–92
75% improvement in ART retention	968–6266	95–225	941–1494	78–503	7–17	66–105
100% improvement in ART retention	1013–6518	93–223	871–1389	97–624	10–23	71–113

Cost estimates are in 2019 US$. ART=antiretroviral therapy. EMOD=EMOD-HIV. LMICs=low-income and middle-income countries. Optima=Optima HIV. Synthesis=HIV Synthesis.

## Discussion

Despite having diverse model structures, assumptions about the health and transmission effects of treatment interruptions, and baseline settings being represented, the EMOD, Optima, and Synthesis models produced comparable estimates for the health benefits and changes in transmission resulting from each additional person-year retained on ART. Transmission reductions, both discounted and undiscounted over a 40-year time horizon, were similar across all three models. Health gains were similar when undiscounted, but different across models when discounted because of different kinetics of changes in mortality as a result of improved retention. These differences probably reflect different assumptions about HIV disease progression and mortality during treatment interruption. Tracing studies have attempted to quantify outcomes among patients lost to follow-up from clinical cohorts, but have struggled to disambiguate patients who died as a result of ART interruption from those who appeared to stop ART as a result of having died from causes other than AIDS, or from treatment failure occurring without treatment interruption. As a result, models have primarily relied on studies of ART-naive cohorts before the treat-all era to develop assumptions about the role of CD4 count, viral load, ageing, and other clinical factors contributing to mortality risk during ART interruptions. Further study of health status during ART interruptions in the treat-all era could help to clarify the contribution of ART interruptions to HIV transmission and burden in sub-Saharan Africa and help HIV programmes to determine when to prioritise retention interventions among competing priorities for HIV care and prevention.

The three models provided different estimates for health benefits and transmission reductions when a retention intervention is offered to all people on ART, reflecting differences in the HIV care continuum across modelled settings. In South Africa, the so-called second 90—ie, the proportion of people diagnosed with HIV who are on ART—constitutes the largest gap in progress towards the 90–90–90 targets.^31^ Accordingly, improved retention had a larger effect in South Africa than in Malawi, which, as of 2017, had surpassed the second 90 target.^32^

Upper-bound costs at which retention interventions could remain cost-effective ranged widely depending on the potential effect of retention in a particular setting, the cost-effectiveness threshold used for the given setting, and the ability to target interventions to people living with HIV who would otherwise have interrupted ART. Previous analyses, before the implementation of treat-all guidelines in sub-Saharan Africa, estimated an upper-bound cost threshold of $10 per patient-year on ART for an intervention that improves retention by 40%. This estimate is similar to the range we estimated using Optima (Malawi) without targeting those most at risk of having interrupted ART. With targeting or a higher cost-effectiveness threshold, or in a setting in which improved retention would have a greater health impact (eg, the range of LMIC settings in sub-Saharan Africa represented within Synthesis), upper-bound costs to improve retention would be higher than $10 per person-year on ART.

Our analysis has several limitations. We assumed that retention interventions would be equally effective at reducing the rate of treatment discontinuation for all people on ART. Studies of specific retention interventions have noted variable effect sizes according to a number of sociodemographic factors, which should be explored further in intervention-specific analyses. We were unable to separate the direct effect of ART retention on the number receiving ART from the indirect effect of changes to HIV incidence and mortality on the number receiving ART. Further analyses separating these effects could be informative for more detailed costing and to understand differences across models. We assumed that ART would be the driver of costs of retention interventions and did not include the population-level effect of improved ART retention on other HIV services such as HIV testing and prevention. We made this assumption because ART is the driver of costs for HIV programmes in sub-Saharan Africa and the effect of improved retention on costs of other services would depend on future policy decisions. For example, reductions in HIV incidence would increase the number of HIV-negative individuals who are able to receive HIV testing and prevention services but, at sufficiently low HIV incidence, these services might be offered less frequently. Finally, our multimodel approach is both a strength and a limitation. Use of models with different structures, assumptions, and baseline settings allowed us to capture the variability of results across sub-Saharan Africa HIV models; however, we did not attempt to standardise individual components of models or systematically assess how particular model attributes affected the estimates provided. Such standardisation and in-depth analysis can help identify the main reasons why model estimates differ. However, performing this type of exercise can run the risk of inducing a so-called groupthink phenomenon and lose the diversity of model structures and assumptions that our analysis intended to capture.

We used a multimodel approach that captures structural model uncertainty and heterogeneity of settings to broadly inform research priorities and regional policy guidelines. For our analysis to be representative of the range of current and future retention interventions, we used a bounding analysis from 0% to 100% precision of targeting those most at risk of interruption. We also used a wide range of cost-effectiveness thresholds. As a result, our analysis carries a wide range of uncertainty and might not provide inference for decision making for some settings—eg, when the cost-effectiveness of a particular intervention falls inside the uncertainty range of upper-bound costs. Decision makers seeking to apply our findings to specific populations and interventions should consider collaborating with modellers to develop models specific to their populations, settings, and interventions of interest to improve accuracy and reduce uncertainty in upper-bound cost estimates. Nevertheless, the effects and upper-bound cost ranges estimated here broadly indicate that the amount that HIV programmes might be willing to invest in retention interventions is similar or substantially higher in the era of treat all than earlier estimates, notwithstanding recent decreases in HIV incidence and mortality. Research on strategies to improve ART retention should be encouraged, especially when it is possible to target those most at risk of ART interruption.

## Data sharing

No new primary data were collected for this study. Source code for each of the three models used in the study is available at the following locations: EMOD: https://github.com/InstituteforDiseaseModeling/EMOD/; Synthesis: https://github-pages.ucl.ac.uk/hiv-synthesis/code.html; and Optima: https://github.com/optimamodel/optima.

## Declaration of interests

Unless otherwise stated, all authors are salaried employees of the institutions to which they are affiliated in the header. AB declares grants from the Bill & Melinda Gates Foundation (BMGF) and the US National Institutes of Health (NIH). LJ declares grants from the United States Agency for International Development (USAID) and BMGF. ANP declares grants from NIH, UK Research and Innovation (UKRI), Wellcome Trust, and BMGF and payment for serving on the HIV Glasgow Congress steering committee. VC declares grants from UKRI, Unitaid, National Institute for Health Research, USAID, UK Research and Innovation Medical Research Council, and BMGF and consulting fees from WHO. GM-R declares grants from BMGF, USAID South Africa, USAID, and Foundation for Innovative New Diagnostics and serving in leadership or fiduciary roles at the WHO, South African Department of Health, and South African National AIDS Council. All other authors declare no competing interests.
