# Supplementary material for: Transmission reduction, health benefits, and upper-bound costs of interventions to improve retention on antiretroviral therapy: a combined analysis of three mathematical models
Source: Lancet Glob Health. 2022 Aug 9;10(9):e1298–306. doi: 10.1016/S2214-109X(22)00310-2 (PMC9380252; doi:10.1016/S2214-109X(22)00310-2)
Supplement: Supplementary appendix [file mmc1.pdf]

THE LANCET
Global Health

Supplementary appendix

This appendix formed part of the original submission and has been peer reviewed. We post it as supplied by the authors.

Supplement to: Bershteyn A, Jamieson L, Kim H-Y, et al. Transmission reduction, health benefits, and upper-bound costs of interventions to improve retention on antiretroviral therapy: a combined analysis of three mathematical models. *Lancet Glob Health* 2022; **10:** e1298–306.

Supplementary Appendix to: **Transmission reduction, health benefits, and upper-bound costs of interventions to improve retention on antiretroviral therapy: a combined analysis of three mathematical models.**

A. Examples of retention interventions and their effect sizes

Numerous studies have tested interventions to improve retention on antiretroviral therapy (ART) among people living with HIV (PLHIV) in Sub-Saharan Africa (SSA). Below we report the effect sizes of a retention interventions in 39 studies spanning 14 SSA countries. Interventions are classified into the categories of community-based service delivery, decentralized care, differentiated care, mHealth, instrumental support, task-shifting, and patient tracing. The studies measured a wide range of effect sizes, suggesting that the intervention type and differences in settings, patient populations, implementation, and study design can lead to different measured outcomes for improvement in retention. For this reason, our modeling study explored a wide range of effectiveness levels for the hypothesized intervention.

Table S1. Measured effect sizes of retention interventions by type, country, and study design.

Intervention type	Year	Country	Study design (sample size)	Intervention	Outcome measurement	Outcome	Ref
Community-based service delivery	2021	Zambia	Prospective cohort study (n=2506)	Four models of care: Community adherence groups, mobile ART, urban adherence groups, home ART.	12-month retention (facility visit between 9-15 months after model entry).	Retention highest in urban adherence groups (95%), followed by community adherence groups (83%), home ART delivery (79%), mobile ART (69%), and standard care (81%).	1
	2019	Zimbabwe	Randomized clinical trial (n=94)	Monthly support groups and weekly visits from community	Increase in self-reported retention in care report for those receiving	Increase in score for intervention participants (3.66 to 3.74, p<0.001), decrease in mean score for	2

				health workers.	support from CATS.	participants in standard of care (3.86 to 3.31, p<0.001).	
	2018	Tanzania	Cluster randomized clinical trial (n=2172)	Delivery of ART by community health workers.	Loss to follow-up (did not return to study facility for study exit assessment or latest viral load not available).	Fewer participants lost to follow-up in standard care group (13.6%) than in the intervention group (18.9%).	3
	2017	Mozambique	Retrospective cohort study (n=2406)	Peer-supported community-level ART distribution.	Retained in care at least 6 months.	Participants in intervention group had greater retention at 12-month and 24-months (99.1% and 97.5%) than participants in the standard care group (89.5% and 82.3%, p<0.0001).	4
	2016	South Africa	Retrospective cohort study (n=8150)	Community-based adherence clubs lead by community health workers and supported by nurse.	Loss to follow-up (no visits in the first 12 weeks prior to study end).	Participation in intervention groups was associated with 67% reduction in loss to follow-up compared with standard care (aHR 0.33, 95% CI: 0.27-0.40).	5
	2015	Kenya	Quasi-experimental<br><br>two-group<br><br>study (n=369)	Community-based patient-defined support groups (microclinics) led by community	Clinic absence (90 more days in the 22-month period after ART initiation).	Intervention participants had one half the rate of clinic absence compared to those in standard care [HR 0.48,	6

				health workers.		95% CI: 0.25-0.92].	
	2014	Mozambique	Retrospective cohort study (n=5729)	Community-level ART distribution and support group.	Retention in care.	Retention at 1 year on ART was 97.7% (95% CI 97.4–98.2); at 2 years, 96.0% (95% CI 95.3–96.6); at 3 years, 93.4% (95% CI 92.3–94.3); and at 4 years, 91.8% (95% CI 90.1–93.2).	7
	2012	Zambia	Retrospective cohort study (n=523)	Community-based support teams of volunteers who provide education, referrals, adherence counseling, defaulter tracing, orphan support.	Retained in care (alive and not lost to follow-up).	Participating in the intervention did not have an effect on retention in care (80%) when compared to standard care (82%, p=0.6).	8
	2012	South Africa	Prospective cohort study (n=66,953)	Community-based adherence support with weekly visits for one month from community-based health workers.	Loss to follow-up (no clinic visits for 180 days or more)	Lower loss to follow-up in intervention group (aHR 0.63, 95% CI: 0.59-0.68).	9
	2011	South Africa	Retrospective cohort study (540)	Community-based adherence support from health workers who provide education, support, and home visits.	Non-retention in case (loss to follow-up or death).	Non-retention in care was lower in intervention group than standard care group (HR 0.62, 95% CI: 0.62-0.68, p=0.001).	10

	2010	Kenya	Cluster randomized clinical study (n=208)	Home visits by community health workers to collect data on symptoms, vital signs, and ART adherence, and dispense one-month supply of medications.	Loss to follow-up at study closure	No significant difference in loss to follow-up between intervention (5.2%) and standard care groups (4.5%, p=1.0).	11
	2007	Mozambique	Randomized clinical trial (n=350)	6-week modified directly observed therapy, delivered daily by peers.	Retention in care 12 months after starting treatment	More participants in intervention group (84.5%) were retained in care than the standard care group (74.3%, OR=1.8, 95% CI: 1.1-3.3).	12
Decentralized care	2015	Kenya	Retrospective cohort study (n=178)	Semi-mobile HIV clinic located near patient homes.	Retention in treatment (ratio of number of scheduled monthly visits attended to total number months in treatment).	Retention did not differ significantly between intervention group (77%) and standard care group (71%, p=0.2).	13
	2013	Lesotho	Retrospective cohort study (n=3747)	Care at health centers led by nurses.	Three-year retention in care (in active follow-up at study end).	Retention did not differ significantly between intervention group (68.7%) and standard care (69.7% p=0.81).	14

	2012	Malawi	Retrospective cohort study (n=15421)	Care provided by mobile teams at peripheral health facilities, nurse-led initiation of ART and clinical monitoring.	Attrition (deaths and loss to follow-up for more than 2 months).	2- year attrition was lower in the intervention group (9.9 per 100-person years) than in the standard care group (20.8 per 100 person years, 95% CI:19.7-22.0).	15
Differentiated care	2018	South Africa	Retrospective cohort study (n=6706)	Community-based adolescent care	Loss to follow-up (at end of study or 5 years on ART, whichever came first)	Fewer participants were lost to follow-up in the intervention group (29.9%) than standard care group (38.9%, aHR 0.60 (95% CI 0.51-0.71); p< 0.0001).	16
	2017	Zimbabwe	Randomized clinical trial (n=334)	Decentralized care and structured support visits by community health workers.	Composite outcome: virally unsuppressed, did not start ART, died, or lost to follow-up (no contact with facility for 6 months and not re-entering care elsewhere 18 months after enrolment).	The proportion of participants with the composite outcome was lower in the intervention group (44%) than the standard care group (58%, aOR 0.50, 95% CI 0.28-0.89, p=0.02).	17
	2017	Malawi	Case-control study (n=617)	Youth-oriented HIV care.	Not retained in care (lost to follow-up, died, or stopped participation).	Fewer not-retained participants in the intervention group (7.9%) than participants in standard of care (35.2%, p<0.01).	18

	2017	South Africa	Retrospective cohort study (n=241)	Adolescent-oriented care.	Retention in care (one clinic visit or pharmacy refill in the prior 6 months).	More participants in intervention group were retained in care (95%) than those participating in standard care (85%, aOR = 8.5; 95% CI 2.3–32.4; p = 0.002).	19
	2016	Kenya	Retrospective cohort study (n=269)	Youth and adolescent-oriented care.	Lost to follow-up during the first 6 months of treatment initiation	Participating in youth-oriented services did not improve retention rates (17.0%) when compared to participants in standard care (16.2%, p=0.77).	20
	2015	Kenya	Retrospective cohort study (n=924)	Youth-oriented HIV care.	Loss to follow-up (absent from HIV treatment clinic for 4 or more months)	Participating in youth-oriented services was not associated with loss to follow-up (aHR 1.09, 95% CI: 0.80–1.56, p=0.56).	21
mHealth	2016	Mozambique	Randomized clinical study (n=830)	Text message reminders.	Retention in care after 12 months of start of treatment	No statistical difference in retention between intervention (93.8%, 95% CI: 90.5-95.7) and standard care (91.0%,95% CI: 87.7-93.4, rate difference-2.8, 95% CI: -0.9-6.4, p=0.139)	22

	2015	South Africa	Randomized clinical study (n=230)	Text message reminders.	Retained in care (completed the study).	Greater proportion of participants completed the study in the intervention group (86.1%) than standard care group (75.7%).	23
	2012	Cameroon	Randomized clinical trial (n=200)	Motivational text messages.	Retention in care after 6 months of care.	No significant difference in proportion of participants retained in the intervention group (79.2%) and standard care group (83.8%, RR 0.95, 95% CI 0.83-1.08, p=0.399).	24
	2010	Kenya	Randomized clinical trial (n=538)	Text message-based support.	Loss to follow-up (unable to reach within 3 months after study end date).	No significant difference in loss to follow-up among participants in the intervention group (6%) and standard care group (10%, RR 1.69, 95% CI: 0.91-3.23, p=0.094).	25
Instrumental support	2018	Uganda	Cluster randomized trial (n= 702)	Child savings account for adolescents.	Attrition (at 24 months after enrollment).	Attrition rate in the intervention group (6.5%) was similar to the standard care group (5.5%).	26
	2017	Mozambique	Cluster randomized clinical trial (n=2004)	Combination intervention strategy (point-of-care testing, accelerated ART	Retention at the diagnosing facility 12 months after diagnosis.	Additional noncash incentives were not associated with improved retention	27

				initiation, SMS health messages and appointment reminders; in addition, conditional noncash financial incentives for linkage and retention)		(55%) when compared to combination intervention without incentives (58%, RR 0.95, 95% CI: 0.79-1.13, p=0.45); fewer participants were retained in the standard care group (44%) when compared to the combination intervention strategy (RR 1.32, 95% CI: 0.79-1.13, p=0.004).	
	2017	Swaziland	Cluster randomized trial (n=2201)	Combination intervention strategy (point-of-care testing, accelerated ART, text message reminders, noncash financial incentives.	Retained in care 12 months after testing.	Higher proportion of participants in intervention group retained in care (66%) than standard care group (45%, RR 1.48, 95% CI: 1.18-1.86, p=0.002)	28
	2014	Chad	Retrospective cohort study (n=509)	Free-of-charge ART.	Loss to follow-up (3 or more months since last visit).	Fewer participants lost to follow up in the intervention group (10%) than standard care group (72.3%, p<0.001).	29
	2014	Uganda	Retrospective cohort study (n=2371)	Food or education support, or both.	Loss to follow-up (no contact 90 or more days after scheduled follow-up, dead, or	More participants lost to follow-up in the food support group (42.1%), fewer participants	30

					transferred elsewhere).	lost to follow in education support group (12.3%), and those who received both interventions (13.7%).	
	2013	Rwanda	Prospective cohort study (n=610)	Daily visit by community health worker, monthly food ration, transportation stipend, accompanied clinic visits by community health workers.	Attrition from treatment during the first year of ART (death, loss to follow-up, or default).	Exposure to the intervention group was strongly associated with a lower risk of attrition (HR, 0.17; 95% CI, .09–.35; p<0.0001)	31
	2011	Cameroon	Retrospective cohort study (n=2920)	Price reduction of ART.	Active in care 15 months of follow-up.	Probability of remaining alive and active in care did not significantly different between the two groups (HR 1.1; 95% CI: 0.9-1.2).	32
Task-shifting	2014	South Africa	Retrospective cohort study (n=5746)	Down-referral to nurse-managed care.	Loss to follow-up (no contact in 6-month period between end of analysis and study end).	Down-referred patients were more likely to be lost to follow-up than not down-referred patients (aHR 1.36, 95% CI: 1.09-1.69).	33
	2013	Malawi	Retrospective cohort study (n=10112)	Nurse-led care.	2-year program attrition (loss to follow-up or death).	Attrition was higher in standard care group when compared to nurse-led group (aIRR 3.03, 95% CI: 2.56-3.59).	34

	2013	South Africa	Prospective cohort study (n=2829)	Adherence clubs led by counselors	Composite outcome of death or loss to follow-up (no contact with clinic for at least 6 months).	Participation in the intervention group reduced death or loss to follow-up (HR 0.43, 95% CI: 0.21-0.91).	35
	2012	Kenya	Retrospective cohort study (n=4958)	One-stop care clinic with interim visits managed by nurses in the first 3 months of ART initiation.	Loss to follow-up (absent from the clinic for at least 3 months).	Participants in the intervention group were less likely to be lost to follow-up than the standard of care group (aHR 0.62; 95% CI: 0.57-0.67).	36
	2012	South Africa	Cluster randomized clinical trial (n= 9252)	Streamlining Tasks and Roles to Expand Treatment and Care for HIV (STRETCH): nurses initiated and prescribe ART.	Retention (alive and in care, with documentation of clinic visit or lab test in previous 6 months) at 12 months after enrollment.	More participants in the intervention group were retained in care (63%) than in the standard care group (58%, RR: 1.10, 95% CI: 1.04-1.16, p<0.001).	37
	2011	South Africa	Retrospective matched cohort study (n=2772)	Down-referral of patients to nurse-managed local primary healthcare clinics for continued monitoring and treatment.	Loss to follow-up (3 or more months late for last scheduled visit).	Down-referred patients were less likely become lost to follow-up (1.4%) than those who were not down-referred (4.2%, aHR 0.3, 95% CI: 0.2–0.6).	38
Patient tracing	2015	Uganda	Prospective (n=256)	Tracking of patients who missed their clinic	Retained in care over 18 months of follow-up.	More participants in the intervention group were retained in	39

				appointment for 8–90 days.		care (39%) than those in standard care group (61%).	

CI: confidence interval; HR: hazard ratio; aHR: adjusted hazard ratio; aIRR: adjusted incident rate ratio; OR: odds ratio; aOR: adjusted odds ratio; RR: relative risk

B. Extended model descriptions

EMOD

Background: EMOD-HIV,[40,41] referred to here as EMOD, [42,43] is an individual-based SSA HIV model that includes an age-structured transmission network with short- and long-term sexual partnerships,[44,45] individual-level HIV disease progression,[40,41] and a detailed continuum of HIV prevention[46,47] and treatment.[48,49] Predictions from EMOD have been systematically compared to study results for prospective validation. The model successfully predicted the outcome of a multi-country community-randomized trial in SSA prior to trial unblinding.[50] It predicted population-level epidemic trends (prevalence, incidence) in multiple countries ahead of release of survey results.[42,51]

Disease progression: Progression of untreated HIV disease is assumed to be age-dependent.[52–56] For example, for an individual infected at age 20, median survival without treatment is 13.1 years (IQR 8.4 – 18.5 years) whereas for survival for an individual infected at age 50, median survival without treatment is 6.3 years (IQR 4.1 – 8.9 years).[52,53,57] After an abrupt drop in CD4 count during acute infection, CD4 count is assumed to decline on a square root scale [58,59] during untreated HIV disease. Three months after infection, median CD4 count is 507 cells/μL (IQR 398 – 613 cells/μL) and at time of death median CD4 count is 19 cells/μL (IQR: 9 – 42 cells/μL).[60]

Effect of ART: Initiation of ART reconstitutes CD4 counts on a square root scale by up to 287 cells/μL over the first three years on ART.[61,62] Survival on ART is assigned in age/sex strata depending on CD4 count, AIDS clinical stage at time of treatment initiation (or re-initiation), and whether or not individuals are adherent to ART.[63–69] Individuals who are adherent to ART are assumed to have a 96% reduction in transmission[70], while individuals who are non-adherent or have interrupted ART are assumed to have no change in their transmission potential.

ART interruptions: The rate of ART interruptions lasting >1 month was 18.7% prior to implementation of Treat All [71–73] and declined to 3.4% per year by 2020 [74–77]. ART interruptions are assumed to result in resumption of untreated HIV progression based on the age and CD4 count at the time of interruption. Because EMOD simulates an age-structured transmission network using the same individuals who experience the above-described disease progression and care continuum, the model captures differences in HIV transmission potential among younger ART-naïve individuals versus older ART-experienced individuals.

Sensitivity analyses: Sensitivity of cost and disability-adjusted life years (DALYs) to different model assumptions about ART retention and re-initiation can be found in earlier published work.[48]

Optima

Background: Optima HIV,[78,79] referred to here as Optima, is a compartmental model with populations disaggregated depending on setting. The model for Malawi is disaggregated by sex, 5-year age groups, and risk (female sex workers, clients of female sex workers, and men who have sex with men) and has been validated by in-country stakeholders. HIV acquisition risk depends on characteristics of the individual (number of sexual partners, number of drug injections) and their partnerships (type of sexual interaction, sexual acts per partner, condom use

(95% risk reduction), male circumcision status (58% reduction), prevention of mother-to-child transmission (PMTCT) status for mother-to-child transmission (90% reduction), pre-exposure prophylaxis (PrEP) (86% reduction adjusted for adherence) and post-exposure prophylaxis (PEP) (51% reduction adjusted for adherence) use, receptive needle-sharing or opiate substitution therapy, and population status (HIV testing, diagnosis, HIV prevalence, un/suppressive ART use (50% reduction for unsuppressive ART, 100% reduction for suppressive ART), and stage of infection).

Disease progression: Progression of untreated HIV disease is defined as transitions through the following categories: acute infection, CD4≥500, CD4 350-499, CD4 200-349, CD4 50-199, and CD4<50

Effect of ART: CD4 count and viral load change at rates depending on ART use and latest reported CD4 count and viral load. Mortality both on and off ART depends on latest reported CD4 count and ART status (un/suppressive), varying between 0.08% per year with CD4>500 on suppressive ART to 32.3% for CD4<50 not on ART.

ART interruptions: Rates of ART interruption are calibrated to balance the annual number diagnosed, initiated on ART, and receiving ART. Prior to 2021, these numbers were entered annually by stakeholders into the Optima input tables by stakeholders. Starting in 2021, the number on treatment was constrained by the proportion of diagnosed PLHIV that remain linked to care, with loss to follow up rates calibrated to match the reported proportional coverage of diagnosed PLHIV in 2020. Individuals who discontinued care are assumed to return to care when they reach CD4<200 via disease progression.

Sensitivity analyses: Sensitivity analyses of cost and DALYs to model parameters can be found in earlier published work.[79]

Synthesis

Background: HIV Synthesis[80–83] is an individual-based HIV model that tracks a simulated population of adults with attributes including age, sex, primary and non-primary condomless sex partners, whether currently a female sex worker, HIV testing, male circumcision status, presence of sexually transmitted infections, and use of PrEP. A series of 22 "setting-scenarios" were generated by sampling several parameter values to represent the range of settings and communities in SSA and to incorporate uncertainty in model assumptions.[84] HIV transmission is simulated between primary partners, and for non-primary partners, HIV acquisition risk depends on the viral load distribution among people of the opposite sex and in age categories determined by age-sex mixing patterns. In HIV-positive people, the model tracks CD4 count, viral load, and ART.

Disease progression: HIV mortality risk is assessed in each three-month period according to CD4 count, viral load, age, and presence of WHO Stage 3 and 4 AIDS-defining conditions.[85]

Effect of ART: The model tracks each HIV-infected individual's ART regimen, ART adherence, and drug resistance mutations. For each drug in the individual's ART regimen, the model calculates the antiviral effect based on the presence of specific drug resistance mutations, drug

potency, and level of adherence. The sum of the antiviral effects determines the impact of the drug regimen on viral load, drug resistance, and CD4 count, and hence risk of AIDS and death.

ART interruptions: The underlying rate of interruption of ART is sampled for each setting scenario from a distribution of 0.2%, 0.4%, 0.8% and 1.5% per 3 months, each with a probability of 0.25 (the realized distribution for the 22 setting scenarios was 18%, 27%, 45%, 9%). The actual rate of interruption depends on several other factors in addition to the underlying rate: presence of current drug toxicity (relative risk = 2, 10, 30, each with probability 0.33 per setting scenario), ART adherence level (relative risk 1.5 if adherence 50-80%, 2 if adherence < 50%; in 25% of setting scenarios these relative rates are increased 2 fold, in 25% of runs they are increased 5 fold), current pregnancy (relative risk 0.01), and more than 1 year from start of ART (relative risk 0.5). In addition, in 20% of setting scenarios there is an effect such that those with recent non-primary condomless sex partners have a 1.5-fold higher risk of interruption. ART interruptions causes a rise in viral load to pre-ART level and a decline in CD4 count towards pre-ART levels.[86] Return to care after interruption occurs at a rate of 10% per 3-month period in 40% of setting scenarios, and at rates of 1%, 5%, 30%, and 60% per 3-month period, each in 15% of setting scenarios respectively. The actual rate that applies for a given person is influenced by (i) the lifetime adherence attribute of person, (ii) whether they have developed an HIV related condition, (iii) pregnancy, and (iv) number of sexual partners, where higher numbers of partners are associated with lower likelihood of re-initiating care. In addition, individuals who remain in care but interrupt ART resume ART at 3-monthly rates of 80%, 85%, 90%, and 95%, each in 25% of setting scenarios respectively. The actual rate is then influenced by (i) whether they have developed an HIV related condition, (ii) whether viral load was measured to be >1000 copies/mL for people for whom the clinic is not aware they have interrupted ART, and (ii) pregnancy. Further details about these assumptions can be found in publications.[87]

Sensitivity analyses: Sensitivity of cost and DALYs to model parameters related to ART can be found in earlier published work.[82,83,85,86]

C. Calculating upper-bound cost with alternative cost-effectiveness thresholds

Upper-bound costs have been calculated for hypothetical interventions with costs that are variable or unknown, such as HIV vaccines,[88] long-acting ART,[89] and long-acting oral PrEP.[90] Upper-bound costs are calculated by determining the net monetary benefit (NMB) of an intervention is positive, when considering the incremental costs and the incremental health benefits multiplied by the cost-effectiveness threshold (CET) for the modeled setting:

$$NMB = CET \times incremental\ DALYs\ averted - incremental\ costs$$

where incremental costs are the cost difference between the intervention and baseline (no intervention) scenarios, and incremental DALYs averted is the difference in DALYs between the intervention and baseline scenarios. Both incremental costs and incremental DALYs are discounted by the same annual discount rate, e.g., 3% per year. Costs and CET use the same currency of 2019 USD. If (NMB) is positive, then the intervention is considered to be cost-effective.

To calculate the upper-bound cost of an intervention, we calculate the maximum possible intervention cost at which NMB does not become negative, i.e., where

$$CET \times incremental\ DALYs\ averted = \ incremental\ costs.$$

Costs include the cost of the retention intervention, as well as the changes to the cost of other HIV services. We assume that ART is the main component of changes to costs of other HIV because it is a cost-driver in SSA HIV programs,[91,92] and because the effects of ART on other program costs would depend on policy decisions. For example, improving ART retention reduces HIV incidence the model projections, which increases the number of HIV-negative individuals in the population who could receive HIV testing and HIV prevention, potentially increasing program costs. However, at sufficiently low HIV incidence, some prevention services may no longer be offered to some populations, and HIV testing might be offered less frequently, which would reduce program costs. Therefore, we calculated incremental costs based on the following equation, with ART cost (Table S2) as the only differential cost component outside of the retention intervention:

$$incremental\ costs\ \approx retention\ interventon\ cost + \\ incremental\ person\text{-}years\ on\ ART \times annual\ ART\ cost$$

Table S2. Annual ART cost

Model	Annual ART cost (2019 USD)	Reference
**EMOD**	$206.75	93
**Optima**	$165.50	94
**Synthesis**	$165.50	95

For maximally targeted retention interventions provided only to people who will interrupt ART, the maximum annual cost per person-year receiving a retention intervention, $I_{targeted}$, is related to the CET in a linear fashion, with the incremental DALYs averted per additional person-years retained on ART (main manuscript, Figure 2a) as the slope, and the annual ART cost as the intercept:

$$\max(I_{targeted}) = \frac{incremental\ DALYs\ averted}{incremental\ PY\ retained\ on\ ART} \times CET - annual\ ART\ cost$$

For untargeted retention interventions provided to all people ART, the maximum annual cost per person-year receiving a retention intervention, $I_{untargeted}$, is also related to the CET in a linear fashion, with the incremental DALYs averted per total person-years on ART (main manuscript, Figure 2a) as the slope, as follows:

$$\max(I_{untargeted}) = \frac{incr.DALYs\ averted}{total\ PY\ on\ ART} \times CET - \frac{incr.PY\ retained\ on\ ART}{total\ PY\ on\ ART} \times annual\ ART\ cost$$

Upper-bound costs with alternative CETs can be calculated using the above equations. For convenience, the slope terms governing the slope (Table S3) and intercept (Table S4) have been calculated. To calculate the upper-bound cost for a retention intervention, multiply a value in Table S3 by the CET and add the corresponding value in Table S4, using the following color-coded equation:

$$upper\text{-}bound\ cost = \boldsymbol{value\ from\ Table\ S3} \times CET + \boldsymbol{value\ from\ Table\ S4}$$

Table S3. Slope of upper-bound cost equation

		Retention interventions for people most-at-risk of interrupting ART			Retention interventions for all people on ART		
**Model**		EMOD	Optima	Synthesis	EMOD	Optima	Synthesis
**Setting**		South Africa	Malawi	SSA LMICs	South Africa	Malawi	SSA LMICs
**Improvement in retention**	25%	$1.88	$0.53	$2.15	$0.06	$0.01	$0.10
	50%	$1.79	$0.53	$2.41	$0.10	$0.03	$0.14
	75%	$1.99	$0.52	$2.21	$0.16	$0.04	$0.15
	100%	$2.07	$0.52	$2.07	$0.20	$0.05	$0.17

Estimates reported in 2019 USD. SSA LMICs: Sub-Saharan African lower-middle income countries.

Table S4. Intercept of upper-bound cost equation

		Retention interventions for people most-at-risk of interrupting ART			Retention interventions for all people on ART		
**Model**		EMOD	Optima	Synthesis	EMOD	Optima	Synthesis
**Setting**		South Africa	Malawi	SSA LMICs	South Africa	Malawi	SSA LMICs
**Improvement in retention**	25%	$206.75	$165.50	$165.50	$6.30	$4.10	$7.76
	50%	$206.75	$165.50	$165.50	$12.10	$8.35	$9.30
	75%	$206.75	$165.50	$165.50	$16.59	$12.74	$11.60
	100%	$206.75	$165.50	$165.50	$19.80	$17.28	$13.49

Estimates reported in 2019 USD. SSA LMICs: Sub-Saharan African lower-middle income countries.

D. ART coverage and number receiving ART

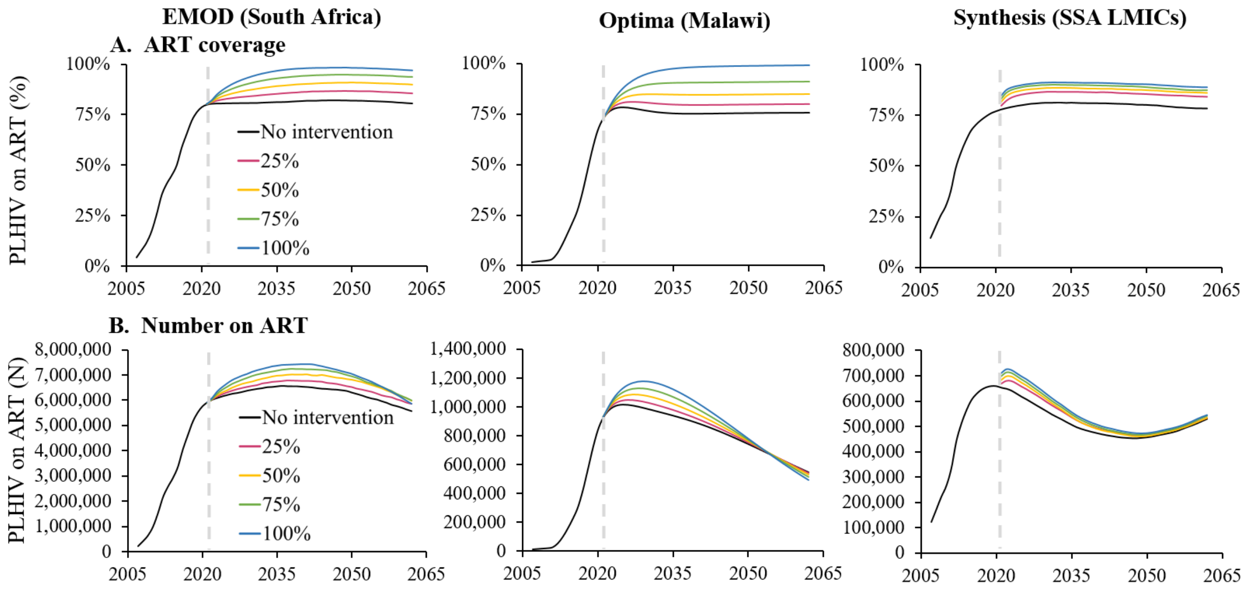

Figure S1. Projections of ART coverage and number on ART with improvements to ART retention. EMOD, Optima HIV, and Synthesis model projections of (A) proportion of PLHIV receiving ART, and (B) number of PLHIV receiving ART.

E. Percent reduction in mortality rate among PLHIV by improving ART retention

Table S5. Percent reduction in mortality rate among PLHIV in the years 2023, 2028, and 2062, corresponding to 1, 5, and 40 years after implementing an improvement in retention.

**Model**		**EMOD**			**Optima**			**Synthesis**		
**Setting**		South Africa			Malawi			SSA LMICs		
**Year**		2023	2028	2062	2023	2028	2062	2023	2028	2062
**Improvement in retention**	25%	2.7%	7.5%	18.6%	1.0%	41.7%	49.7%	25.4%	34.3%	35.7%
	50%	3.1%	15.1%	45.9%	2.0%	46.8%	59.9%	37.1%	44.7%	41.3%
	75%	5.1%	26.1%	68.7%	2.9%	52.0%	72.1%	44.7%	51.7%	40.8%
	100%	6.4%	34.5%	89.1%	3.9%	57.7%	87.4%	51.7%	56.9%	42.9%

F. References

1 Nichols BE, Cele R, Jamieson L, *et al.* Community-based delivery of HIV treatment in Zambia: costs and outcomes. *AIDS* 2021; **35**: 299–306.

2 Willis N, Milanzi A, Mawodzeke M, *et al.* Effectiveness of community adolescent treatment supporters (CATS) interventions in improving linkage and retention in care, adherence to ART and psychosocial well-being: a randomised trial among adolescents living with HIV in rural Zimbabwe. *BMC Public Health* 2019; **19**: 117.

3 Geldsetzer P, Francis JM, Sando D, *et al.* Community delivery of antiretroviral drugs: A non-inferiority cluster-randomized pragmatic trial in Dar es Salaam, Tanzania. *PLOS Med* 2018; **15**: e1002659.

4 Decroo T, Telfer B, Dores CD, *et al.* Effect of Community ART Groups on retention-in-care among patients on ART in Tete Province, Mozambique: a cohort study. *BMJ Open* 2017; **7**: e016800.

5 Grimsrud A, Lesosky M, Kalombo C, Bekker L-G, Myer L. Implementation and Operational Research: Community-Based Adherence Clubs for the Management of Stable Antiretroviral Therapy Patients in Cape Town, South Africa A Cohort Study. *JAIDS J Acquir Immune Defic Syndr* 2016; **71**: e16–23.

6 Hickey MD, Salmen CR, Omollo D, *et al.* Implementation and Operational Research: Pulling the Network Together Quasiexperimental Trial of a Patient-Defined Support Network Intervention for Promoting Engagement in HIV Care and Medication Adherence on Mfangano Island, Kenya. *JAIDS J Acquir Immune Defic Syndr* 2015; **69**: e127–34.

7 Decroo T, Koole O, Remartinez D, *et al.* Four-year retention and risk factors for attrition among members of community ART groups in Tete, Mozambique. *Trop Med Int Health* 2014; **19**: 514–21.

8 Estopinal CB, Dijk JH van, Sitali S, *et al.* Availability of Volunteer-Led Home-Based Care System and Baseline Factors as Predictors of Clinical Outcomes in HIV-Infected Patients in Rural Zambia. *PLOS ONE* 2012; **7**: e49564.

9 Fatti G, Meintjes G, Shea J, Eley B, Grimwood A. Improved Survival and Antiretroviral Treatment Outcomes in Adults Receiving Community-Based Adherence Support: 5-Year Results From a Multicentre Cohort Study in South Africa. *JAIDS J Acquir Immune Defic Syndr* 2012; **61**: e50–8.

10 Igumbor JO, Scheepers E, Ebrahim R, Jason A, Grimwood A. An evaluation of the impact of a community-based adherence support programme on ART outcomes in selected government HIV treatment sites in South Africa. *AIDS Care* 2011; **23**: 231–6.

11 Selke HM, Kimaiyo S, Sidle JE, *et al.* Task-Shifting of Antiretroviral Delivery From Health Care Workers to Persons Living With HIV/AIDS: Clinical Outcomes of a Community-Based Program in Kenya. *JAIDS J Acquir Immune Defic Syndr* 2010; **55**: 483–90.

12 Pearson CR, Micek MA, Simoni JM, *et al.* Randomized Control Trial of Peer-Delivered, Modified Directly Observed Therapy for HAART in Mozambique. *JAIDS J Acquir Immune Defic Syndr* 2007; **46**: 238–44.

13 Gorman SE, Martinez JM, Olson J. An assessment of HIV treatment outcomes among utilizers of semi-mobile clinics in rural Kenya. *AIDS Care* 2015; **27**: 665–8.

14 Labhardt ND, Keiser O, Sello M, *et al.* Outcomes of antiretroviral treatment programmes in rural Lesotho: health centres and hospitals compared. *J Int AIDS Soc* 2013; **16**: 18616.

15 McGuire M, Pinoges L, Kanapathipillai R, *et al.* Treatment Initiation, Program Attrition and Patient Treatment Outcomes Associated with Scale-Up and Decentralization of HIV Care in Rural Malawi. *PLoS ONE* 2012; **7**: e38044.

16 Fatti G, Jackson D, Goga AE, *et al.* The effectiveness and cost-effectiveness of community-based support for adolescents receiving antiretroviral treatment: an operational research study in South Africa. *J Int AIDS Soc* 2018; **21**: e25041.

17 Ferrand RA, Simms V, Dauya E, *et al.* The effect of community-based support for caregivers on the risk of virological failure in children and adolescents with HIV in Harare, Zimbabwe (ZENITH): an open-label, randomised controlled trial. *Lancet Child Adolesc Health* 2017; **1**: 175–83.

18 MacKenzie RK, Lettow M, Gondwe C, *et al.* Greater retention in care among adolescents on antiretroviral treatment accessing “Teen Club” an adolescent-centred differentiated care model compared with standard of care: a nested case–control study at a tertiary referral hospital in Malawi. *J Int AIDS Soc* 2017; **20**. DOI:10.1002/jia2.25028.

19 Zanoni BC, Sibaya T, Cairns C, Lammert S, Haberer JE. Higher retention and viral suppression with adolescent-focused HIV clinic in South Africa. *PLOS ONE* 2017; **12**: e0190260.

20 Teasdale CA, Alwar T, Chege D, Fayorsey R, Hawken MP, Abrams EJ. Impact of Youth and Adolescent Friendly Services on Retention of 10–24-Year-Olds in HIV Care and Treatment Programs in Nyanza, Kenya. *JAIDS J Acquir Immune Defic Syndr* 2016; **71**: e56–9.

21 Ojwang' V, Penner J, Blat C, Agot K, Bukusi E, Cohen C. Loss to follow-up among youth accessing outpatient HIV care and treatment services in Kisumu, Kenya. *AIDS Care* 2016; **28**: 500–7.

22 Joseph Davey D, Nhavoto JA, Augusto O, *et al.* SMSaúde: Evaluating Mobile Phone Text Reminders to Improve Retention in HIV Care for Patients on Antiretroviral Therapy in Mozambique. *JAIDS J Acquir Immune Defic Syndr* 2016; **73**: e23–30.

23 Orrell C, Cohen K, Mauff K, Bangsberg DR, Maartens G, Wood R. A Randomized Controlled Trial of Real-Time Electronic Adherence Monitoring With Text Message Dosing Reminders in People Starting First-Line Antiretroviral Therapy. *JAIDS J Acquir Immune Defic Syndr* 2015; **70**: 495–502.

24 Mbuagbaw L, Thabane L, Ongolo-Zogo P, *et al.* The Cameroon Mobile Phone SMS (CAMPS) Trial: A Randomized Trial of Text Messaging versus Usual Care for Adherence to Antiretroviral Therapy. *PLoS ONE* 2012; **7**: e46909.

25 Lester RT, Ritvo P, Mills EJ, *et al.* Effects of a mobile phone short message service on antiretroviral treatment adherence in Kenya (WelTel Kenya1): a randomised trial. *The Lancet* 2010; **376**: 1838–45.

26 Bermudez LG, Ssewamala FM, Neilands TB, *et al.* Does Economic Strengthening Improve Viral Suppression Among Adolescents Living with HIV? Results From a Cluster Randomized Trial in Uganda. *AIDS Behav* 2018; **22**: 3763–72.

27 Elul B, Lamb MR, Lahuerta M, *et al.* A combination intervention strategy to improve linkage to and retention in HIV care following diagnosis in Mozambique: A cluster-randomized study. *PLOS Med* 2017; **14**: e1002433.

28 McNairy ML, Lamb MR, Gachuhi AB, *et al.* Effectiveness of a combination strategy for linkage and retention in adult HIV care in Swaziland: The Link4Health cluster randomized trial. *PLOS Med* 2017; **14**: e1002420.

29 Djarma O, Nguyen Y, Renois F, Djimassal A, Banisadr F, Andreoletti L. Continuous free access to HAART could be one of the potential factors impacting on loss to follow-up in HAART-eligible patients living in a resource-limited setting: N'djamena, Chad. *Trans R Soc Trop Med Hyg* 2014; **108**: 735–8.

30 Stella-Talisuna A, Bilcke J, Colebunders R, Beutels P. Cost-Effectiveness of Socioeconomic Support as Part of HIV Care for the Poor in an Urban Community-Based Antiretroviral Program in Uganda. *JAIDS J Acquir Immune Defic Syndr* 2014; **67**: e76–83.

31 Franke MF, Kaigamba F, Socci AR, *et al.* Improved Retention Associated With Community-Based Accompaniment for Antiretroviral Therapy Delivery in Rural Rwanda. *Clin Infect Dis* 2013; **56**: 1319–26.

32 Mosoko JJ, Akam W, Weidle PJ, *et al.* Retention in an antiretroviral therapy programme during an era of decreasing drug cost in Limbe, Cameroon. *J Int AIDS Soc* 2011; **14**: 32.

33 Grimsrud A, Kaplan R, Bekker L-G, Myer L. Outcomes of a nurse-managed service for stable HIV-positive patients in a large South African public sector antiretroviral therapy programme. *Trop Med Int Health* 2014; **19**: 1029–39.

34 McGuire M, Ben Farhat J, Pedrono G, *et al.* Task-Sharing of HIV Care and ART Initiation: Evaluation of a Mixed-Care Non-Physician Provider Model for ART Delivery in Rural Malawi. *PLoS ONE* 2013; **8**: e74090.

35 Luque-Fernandez MA, Cutsem GV, Goemaere E, *et al.* Effectiveness of Patient Adherence Groups as a Model of Care for Stable Patients on Antiretroviral Therapy in Khayelitsha, Cape Town, South Africa. *PLOS ONE* 2013; **8**: e56088.

36 Braitstein P, Siika A, Hogan J, *et al.* A clinician-nurse model to reduce early mortality and increase clinic retention among high-risk HIV-infected patients initiating combination antiretroviral treatment. *J Int AIDS Soc* 2012; **15**: 7–7.

37 Fairall L, Bachmann MO, Lombard C, *et al.* Task shifting of antiretroviral treatment from doctors to primary-care nurses in South Africa (STRETCH): a pragmatic, parallel, cluster-randomised trial. *The Lancet* 2012; **380**: 889–98.

38 Brennan AT, Long L, Maskew M, *et al.* Outcomes of stable HIV-positive patients down-referred from a doctor-managed antiretroviral therapy clinic to a nurse-managed primary health clinic for monitoring and treatment. *AIDS* 2011; **25**: 2027–36.

39 Nakiwogga-Muwanga A, Musaazi J, Katabira E, Worodria W, Talisuna SA, Colebunders R. Patients who return to care after tracking remain at high risk of attrition: experience from a large HIV clinic, Uganda. *Int J STD AIDS* 2015; **26**: 42–7.

40 Bershteyn A, Klein DJ, Wenger E, Eckhoff PA. Description of the EMOD-HIV Model v0.7. *ArXiv12063720 Q-Bio Stat* 2012; published online June 16. http://arxiv.org/abs/1206.3720 (accessed April 1, 2018).

41 Bershteyn A, Gerardin J, Bridenbecker D, *et al.* Implementation and applications of EMOD, an individual-based multi-disease modeling platform. *Pathog Dis* 2018; **76**. DOI:10.1093/femspd/fty059.

42 Eaton JW, Bacaër N, Bershteyn A, *et al.* Assessment of epidemic projections using recent HIV survey data in South Africa: a validation analysis of ten mathematical models of HIV epidemiology in the antiretroviral therapy era. *Lancet Glob Health* 2015; **3**: e598–608.

43 Jewell BL, Balzer LB, Clark TD, *et al.* Predicting HIV Incidence in the SEARCH Trial: A Mathematical Modeling Study. *JAIDS J Acquir Immune Defic Syndr* 2021; **87**: 1024–31.

44 Bershteyn A, Klein DJ, Eckhoff PA. Age-dependent partnering and the HIV transmission chain: a microsimulation analysis. *J R Soc Interface* 2013; **10**: 20130613.

45 Bershteyn A, Mutai KK, Akullian AN, Klein DJ, Jewell BL, Mwalili SM. The influence of mobility among high-risk populations on HIV transmission in Western Kenya. *Infect Dis Model* 2018; **3**: 97–106.

46 Mudimu E, Peebles K, Mukandavire Z, *et al.* Individual and community-level benefits of PrEP in western Kenya and South Africa: Implications for population prioritization of PrEP provision. *PLoS ONE* 2020; **15**. DOI:10.1371/journal.pone.0244761.

47 Klein DJ, Eckhoff PA, Bershteyn A. Targeting HIV services to male migrant workers in southern Africa would not reverse generalized HIV epidemics in their home communities: a mathematical modeling analysis. *Int Health* 2015; **7**: 107–13.

48 Klein DJ, Bershteyn A, Eckhoff PA. Dropout and re-enrollment: implications for epidemiological projections of treatment programs. *AIDS Lond Engl* 2014; **28 Suppl 1**: S47-59.

49 Sharma M, Mudimu E, Simeon K, *et al.* Cost-effectiveness of point-of-care testing with task-shifting for HIV care in South Africa: a modelling study. *Lancet HIV* 2021; **8**: e216–24.

50 Jewell BL, Balzer LB, Clark TD, *et al.* Predicting HIV Incidence in the SEARCH Trial: A Mathematical Modeling Study. *J Acquir Immune Defic Syndr 1999* 2021; **87**: 1024–31.

51 Sachathep K, Radin E, Hladik W, *et al.* Population-Based HIV Impact Assessments Survey Methods, Response, and Quality in Zimbabwe, Malawi, and Zambia. *JAIDS J Acquir Immune Defic Syndr* 2021; **87**: S6.

52 Todd J, Glynn JR, Marston M, *et al.* Time from HIV seroconversion to death: a collaborative analysis of eight studies in six low and middle-income countries before highly active antiretroviral therapy. *AIDS* 2007; **21**: S55-S63 10.1097/01.aids.0000299411.75269.e8.

53 Bygrave H, Mtangirwa J, Ncube K, Ford N, Kranzer K, Munyaradzi D. Antiretroviral Therapy Outcomes among Adolescents and Youth in Rural Zimbabwe. *PLoS ONE* 2012; **7**: e52856.

54 Babiker AG, Peto T, Porter K, Walker AS, Darbyshire JH. Age as a determinant of survival in HIV infection. *J Clin Epidemiol* 2001; **54**: S16–21.

55 Porter K, Babiker A, Bhaskaran K, *et al.* Determinants of survival following HIV-1 seroconversion after the introduction of HAART. *Lancet Lond Engl* 2003; **362**: 1267–74.

56 Babiker A, Darby S, De Angelis D, *et al.* Time from HIV-1 seroconversion to AIDS and death before widespread use of highly-active antiretroviral therapy: a collaborative re-analysis. *LANCET* 2000; **355**: 1131–7.

57 Bershteyn A, Klein DJ, Eckhoff PA. Age-targeted HIV treatment and primary prevention as a 'ring fence' to efficiently interrupt the age patterns of transmission in generalized epidemic settings in South Africa. *Int Health* 2016; **8**: 277–85.

58 Pantazis N, Porter K, Costagliola D, *et al.* Temporal trends in prognostic markers of HIV-1 virulence and transmissibility: an observational cohort study. *Lancet HIV* 2014; **1**: e119–26.

59 Jarrin I, Pantazis N, Dalmau J, *et al.* Does rapid HIV disease progression prior to combination antiretroviral therapy hinder optimal CD4+ T-cell recovery once HIV-1 suppression is achieved? *AIDS Lond Engl* 2015; **29**: 2323–33.

60 Holmes CB, Wood R, Badri M, *et al.* CD4 decline and incidence of opportunistic infections in Cape Town, South Africa: implications for prophylaxis and treatment. *J Acquir Immune Defic Syndr 1999* 2006; **42**: 464–9.

61 Picat M-Q, Lewis J, Musiime V, *et al.* Predicting Patterns of Long-Term CD4 Reconstitution in HIV-Infected Children Starting Antiretroviral Therapy in Sub-Saharan Africa: A Cohort-Based Modelling Study. *PLOS Med* 2013; **10**: e1001542.

62 Bershteyn A, Klein D. STI and HIV Model Introduction, 1st edn. Seattle, WA: Institute for Disease Modeling, 2015.

63 May M, Boulle A, Phiri S, *et al.* Prognosis of patients with HIV-1 infection starting antiretroviral therapy in sub-Saharan Africa: a collaborative analysis of scale-up programmes. *The Lancet* 2010; **376**: 449–57.

64 Pujades-Rodríguez M, Balkan S, Arnould L, Brinkhof MAW, Calmy A. Treatment Failure and Mortality Factors in Patients Receiving Second-Line HIV Therapy in Resource-Limited Countries. *JAMA J Am Med Assoc* 2010; **304**: 303–12.

65 Egger M, Ekouevi DK, Williams C, *et al.* Cohort Profile: The international epidemiological databases to evaluate AIDS (IeDEA) in sub-Saharan Africa. *Int J Epidemiol* 2011; published online May 18. DOI:10.1093/ije/dyr080.

66 Babiker AG, Emery S, Fätkenheuer G, *et al.* Considerations in the rationale, design and methods of the Strategic Timing of AntiRetroviral Treatment (START) study. *Clin Trials Lond Engl* 2013; **10**: S5–36.

67 May MT, Gompels M, Delpech V, *et al.* Impact on life expectancy of HIV-1 positive individuals of CD4+ cell count and viral load response to antiretroviral therapy. *AIDS Lond Engl* 2014; **28**: 1193–202.

68 Johnson LF, Keiser O, Fox MP, *et al.* Life expectancy trends in adults on antiretroviral treatment in South Africa. *AIDS Lond Engl* 2016; **30**: 2545–50.

69 Wandeler G, Johnson LF, Egger M. Trends in life expectancy of HIV-positive adults on antiretroviral therapy across the globe: comparisons with general population. *Curr Opin HIV AIDS* 2016; **11**: 492–500.

70 Cohen MS, Chen YQ, McCauley M, *et al.* Prevention of HIV-1 infection with early antiretroviral therapy. *N Engl J Med* 2011; **365**: 493–505.

71 Sikazwe I, Eshun-Wilson I, Sikombe K, *et al.* Retention and viral suppression in a cohort of HIV patients on antiretroviral therapy in Zambia: Regionally representative estimates using a multistage-sampling-based approach. *PLoS Med* 2019; **16**: e1002811.

72 Fox MP, Bor J, Brennan AT, *et al.* Estimating retention in HIV care accounting for patient transfers: A national laboratory cohort study in South Africa. *PLOS Med* 2018; **15**: e1002589.

73 Gosset A, Protopopescu C, Larmarange J, *et al.* Retention in Care Trajectories of HIV-Positive Individuals Participating in a Universal Test-and-Treat Program in Rural South Africa (ANRS 12249 TasP Trial). *JAIDS J Acquir Immune Defic Syndr* 2019; **80**: 375–85.

74 Bor J, Fox MP, Rosen S, *et al.* Treatment eligibility and retention in clinical HIV care: A regression discontinuity study in South Africa. *PLOS Med* 2017; **14**: e1002463.

75 Lopes J, Grimwood A, Ngorima-Mabhena N, *et al.* Out-of-Facility Multimonth Dispensing of Antiretroviral Treatment: A Pooled Analysis using Individual Patient Data from Cluster-Randomized Trials in Southern Africa. *JAIDS J Acquir Immune Defic Syndr* 2021; published online Sept 30. DOI:10.1097/QAI.0000000000002797.

76 Liu L, Christie S, Munsamy M, *et al.* Expansion of a national differentiated service delivery model to support people living with HIV and other chronic conditions in South Africa: a descriptive analysis. *BMC Health Serv Res* 2021; **21**: 1–8.

77 Cassidy T, Grimsrud A, Keene C, *et al.* Twenty-four-month outcomes from a cluster-randomized controlled trial of extending antiretroviral therapy refills in ART adherence clubs. *J Int AIDS Soc* 2020; **23**: e25649.

78 Kerr CC, Stuart RM, Gray RT, *et al.* Optima: A Model for HIV Epidemic Analysis, Program Prioritization, and Resource Optimization. *J Acquir Immune Defic Syndr 1999* 2015; **69**: 365–76.

79 Kelly SL, Martin-Hughes R, Stuart RM, *et al.* The global Optima HIV allocative efficiency model: targeting resources in efforts to end AIDS. *Lancet HIV* 2018; **5**: e190–8.

80 Harlow AF, Bor J, Brennan AT, *et al.* Impact of Viral Load Monitoring on Retention and Viral Suppression: A Regression Discontinuity Analysis of South Africa’s National Laboratory Cohort. *Am J Epidemiol* 2020; **189**: 1492–501.

81 Cambiano V, Ford D, Mabugu T, *et al.* Assessment of the Potential Impact and Cost-effectiveness of Self-Testing for HIV in Low-Income Countries. *J Infect Dis* 2015; **212**: 570–7.

82 Phillips AN, Bansi-Matharu L, Venter F, *et al.* Updated assessment of risks and benefits of dolutegravir versus efavirenz in new antiretroviral treatment initiators in sub-Saharan Africa: modelling to inform treatment guidelines. *Lancet HIV* 2020; **7**: e193–200.

83 Phillips AN, Venter F, Havlir D, *et al.* Risks and benefits of dolutegravir-based antiretroviral drug regimens in sub-Saharan Africa: a modelling study. *Lancet HIV* 2019; **6**: e116–27.

84 Jewell BL, Mudimu E, Stover J, *et al.* Potential effects of disruption to HIV programmes in sub-Saharan Africa caused by COVID-19: results from multiple mathematical models. *Lancet HIV* 2020; **7**: e629–40.

85 Phillips AN, Cambiano V, Nakagawa F, *et al.* Cost-effectiveness of public-health policy options in the presence of pretreatment NNRTI drug resistance in sub-Saharan Africa: a modelling study. *Lancet HIV* 2018; **5**: e146–54.

86 Phillips A, Shroufi A, Vojnov L, *et al.* Sustainable HIV treatment in Africa through viral-load-informed differentiated care. *Nature* 2015; **528**: S68–76.

87 Phillips AN, Bershteyn A, Revill P, *et al.* Cost-effectiveness of easy-access, risk-informed oral pre-exposure prophylaxis in HIV epidemics in sub-Saharan Africa: a modelling study. *Lancet HIV* 2022; **9**: e353–62.

88 Leelahavarong P, Teerawattananon Y, Werayingyong P, *et al.* Is a HIV vaccine a viable option and at what price? An economic evaluation of adding HIV vaccination into existing prevention programs in Thailand. *BMC Public Health* 2011; **11**: 534.

89 Culhane J, Sharma M, Wilson K, *et al.* Modeling the health impact and cost threshold of long-acting ART for adolescents and young adults in Kenya. *EClinicalMedicine* 2020; **25**: 100453.

90 Kirtane AR, Abouzid O, Minahan D, *et al.* Development of an oral once-weekly drug delivery system for HIV antiretroviral therapy. *Nat Commun* 2018; **9**. DOI:10.1038/s41467-017-02294-6.

91 Meyer-Rath G, van Rensburg C, Chiu C, Leuner R, Jamieson L, Cohen S. The per-patient costs of HIV services in South Africa: Systematic review and application in the South African HIV Investment Case. *PLoS ONE* 2019; **14**: e0210497.

92 Armstrong R, Campbell White A, Chinyamuchiko P, Chizimbi S, Hamm Rush S, Poku NK. Inclusive engagement for health and development or 'political theatre': results from case studies examining mechanisms for country ownership in Global Fund processes in Malawi, Tanzania and Zimbabwe. *Glob Health* 2019; **15**: 34.

93 Meyer-Rath G, Johnson LF, Pillay Y, *et al.* Changing the South African national antiretroviral therapy guidelines: The role of cost modelling. *PLOS ONE* 2017; **12**: e0186557.

94 Vyas S, Songo J, Guinness L, *et al.* Assessing the costs and efficiency of HIV testing and treatment services in rural Malawi: implications for future "test and start" strategies. *BMC Health Serv Res* 2020; **20**: 1–11.

95 Phillips AN, Bansi-Matharu L, Cambiano V, *et al.* The potential role of long-acting injectable cabotegravir–rilpivirine in the treatment of HIV in sub-Saharan Africa: a modelling analysis. *Lancet Glob Health* 2021; **9**: e620–7.
